# The Interplay between Medical Plants and Gut Microbiota in Cancer

**DOI:** 10.3390/nu15153327

**Published:** 2023-07-26

**Authors:** Santino Caserta, Claudia Genovese, Nicola Cicero, Valeria Toscano, Sebastiano Gangemi, Alessandro Allegra

**Affiliations:** 1 Division of Hematology, Department of Human Pathology in Adulthood and Childhood “Gaetano Barresi”, University of Messina, Via Consolare Valeria, 98125 Messina, Italy; 132588@polime.it (S.C.); aallegra@unime.it (A.A.); 2 National Research Council, Institute for Agriculture and Forestry Systems in the Mediterranean, Via Empedocle 58, 95128 Catania, Italy; valeria.toscano@cnr.it; 3 Department of Biomedical, Dental, Morphological and Functional Imaging Sciences, University of Messina, Via Consolare Valeria, 98125 Messina, Italy; ncicero@unime.it; 4 Allergy and Clinical Immunology Unit, Department of Clinical and Experimental Medicine, University of Messina, Via Consolare Valeria, 98125 Messina, Italy; gangemis@unime.it

**Keywords:** cancer, gut microbiota, medical plants, nobiletin, baicalein, evodiamine, resveratrol, daidzein

## Abstract

The gut microbiota is a dynamic community of bacteria distributed in the gastroenteric tract and changes in response to diseases, diet, use of antibiotics and probiotics, hygiene status, and other environmental factors. Dysbiosis, a disruption of the normal crosstalk between the host and the microbes, is associated with obesity, diabetes, cancer, and cardiovascular diseases, is linked to a reduction of anti-inflammatory bacteria like *Lactobacillus* and *Roseburia*, and to an increase in the growth of proinflammatory species like *Ruminococcus gnavus* and *Bacteroidetes*. Some plants possess anticancer properties and various studies have reported that some of these are also able to modulate the gut microbiota. The aim of this work is to evaluate the crucial relationship between medical plants and gut microbiota and the consequences on the onset and progression of cancer. In vivo studies about hematological malignancies showed that beta-glucans tie to endogenous antibeta glucan antibodies and to iC3b, an opsonic fragment of the central complement protein C3, leading to phagocytosis of antibody-targeted neoplastic cells and potentiation of the cytotoxic activity of the innate immune system if administered together with monoclonal antibodies. In conclusion, this review suggests the potential use of medical plants to improve gut dysbiosis and assist in the treatment of cancer.

## 1. Medical Plants and Their Benefits

**Figure 1:**
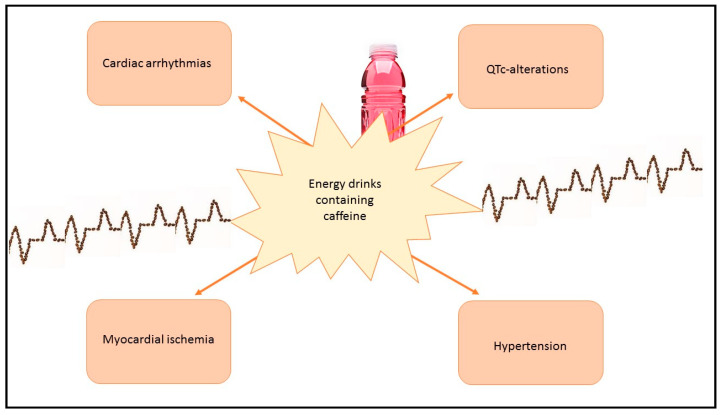
Energy drinks are stimulating beverages containing caffeine that increase the risk of cardiovascular conditions like cardiac arrhythmias, QTc-alterations, myocardial ischemia, and hypertension, and cause a general state of tissue inflammation and oxidative stress which could predispose to cancerogenesis.

Medical plants are those plants that have therapeutic properties or exert a beneficial pharmacological effect on humans. They have long been used and are still used in traditional medicine and worldwide ethnomedicine. Medical plants are a reservoir of biologically active compounds, such as phenolic acids, flavonoids, tannins, alkaloids, coumarins, terpenoids, and organosulfur compounds. A wide spectrum of bioactivities is exhibited by these compounds, such as antimicrobial, antioxidant, anti-inflammatory, and anticancer [1,2,3,4,5]. A diet rich in vegetables has several benefits in terms of health promotion and modulation of the gut microbiota; it is linked to a reduction in systemic and local intestinal inflammation and to a lower risk of diseases like type 2 diabetes and colorectal cancer. Although the first evidence of grain cultivation is very far on the history timeline, some traditional customs are still alive. In India, agriculture spread very early and people developed different habits connected to food, including the main meals in the form of a large round platter called thali with bread or rice and various smaller bowls called katori that separate curry or condiments, and the use of food as a medicine, according to the Ayurvedic system. The Ayurvedic system is based on small portions of food with a balanced content of the six major flavors (sour, bitter, sweet, salty, astringent, and pungent) and it is connected to the idea that a variety of flavors means the presence of different bioactive compounds with a positive impact on human health, even if contained in small quantity, such as minerals and vitamins. Polyphenols are a group of bioactive compounds, including flavonoids like anthocyanins, isoflavones, lignans, stilbenes, and tannins. Anthocyanins, contained mostly in some fruits and vegetables like eggplant, blueberries, and certain carrots and potatoes, are used in the food industry as water-soluble and nontoxic colorants; in fact, most foods containing them are purple, red, or blue with anti-inflammatory and antioxidant properties. The major part of them are not absorbed in the blood throughout the small intestine and reach the colon, where they reduce the inflammation-related oxidative stress on the gut microbes, give bacteria a carbon source for their metabolism with the result of the growth of specific microbes, and the formation of metabolites with antimicrobial action on pathogens like *Escherichia coli* [6,7,8,9]. Glucoraphanin was first described as an antibiotic in the middle of the nineteenth century and then for its anticancer properties; this substance is the precursor of sulforaphane and can be isolated from broccoli, in particular, in the flower buds and the aerial portions of the plant. Broccoli-based preparations were shown to accelerate the excretion of air pollutants and carcinogenetic food contaminants. A randomized placebo-controlled clinical trial used a broccoli sprout beverage administered at the doses of 600 µmol glucoraphanin and 40 µmol sulforaphane for ten days with the results of a better detoxication of benzene compared to the control group [10,11,12,13,14,15,16]. Studies about vascular plants, characterized by lignified tissues named the xylem which conduct water and minerals throughout the plant, showed that there are 391,000 species of them supporting about 1026 bacterial cells. These plant bacterial communities are an important source of airborne microbes and are associated with a greater diversity of gut microbiota. A Scandinavian research study demonstrated that people living in areas with many species of plants have different skin bacteria and a lower risk of atopic sensitization compared to people living in areas with few species of plants. Furthermore, research showed that animals, when exposed to more different plants, have a more different gut microbiota, as observed between rural and urban white-crowned sparrows. In detail, according to the biodiversity hypothesis, it was evidenced that rural white-crowned sparrows that are in contact with a lot of different types of plants have a more variegated gut microbiota and a better immune response compared to that of urban sparrows [17]. Among substances capable of modifying gut microbiota, there are energy drinks, which are stimulating beverages containing molecules like guarana and caffeine and are recognized as physical and mental enhancers. In the last few years, their consumption was augmented, in particular in young people who consume them together with alcoholic drinks or drugs. Different clinical studies that have been related to energy-drink consumption have shown an increase in cardiovascular pathological conditions, such as cardiac arrhythmias, QTc-alterations, myocardial ischemia, and hypertension. The most common metabolite of energy drinks is caffeine, a molecule that activates the sympathetic nervous system by acting as an antagonist of adenosine and leading to a stimulation of sympathomimetic actions and to an increase in blood pressure; this effect depends also on the habitual intake of caffeine of individuals because in vivo experiments showed that habitual coffee drinkers had no effect or less effect on their blood pressure compared with caffeine-withdrawn subjects. All of the possible effects of energy drinks reflect a general state of inflammation and oxidative stress which could predispose to cancerogenesis (Figure 1) [18].

More and more evidence is found in the literature about the healthy properties of molecules contained in plants and it could represent the point of start for future and more specific research in this field.

## 2. Relationship between Medical Plants, Gut Microbiota, and Cancer

**Figure 2:**
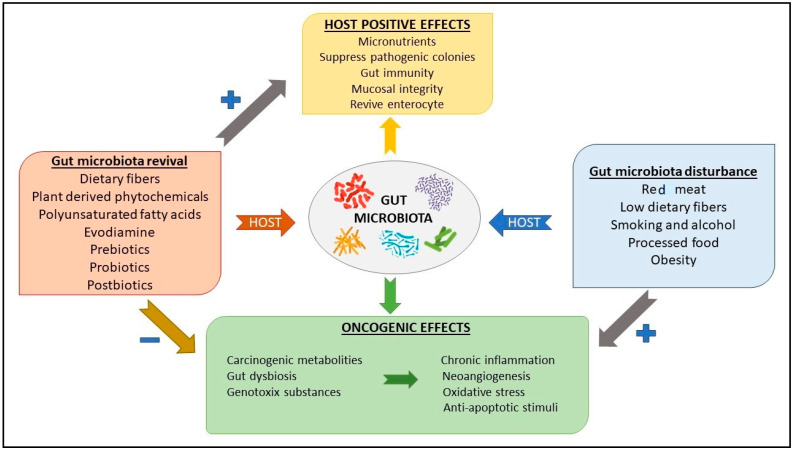
Diet-related molecules reviving gut microbiota like dietary fibers, evodiamine, and pre- and probiotics can have a positive effect on the human organism (host) and prevent cancerogenesis, while dietary factors causing gut dysbiosis like red meat, smoking, and alcohol lead to an increased risk of tumor onset and progression.

The gut microbiota is a dynamic community made up of trillions of bacteria distributed in the gastroenteric tract from the mouth to the anus with pivotal functions in intestinal homeostasis and digestion; it consists of microbes from five phyla: *Firmicutes*, *Bacteroidetes*, *Actinobacteria*, *Verrucomicrobia*, and *Proteobacteria*, with the first two constituting more than 90% of the total population. The gut microbiota changes in response to diseases, diet, use of antibiotics and probiotics, hygiene status, and other environmental factors; for example, a diet rich in fiber is linked to higher microbial diversity and to a smaller content in *Enterobacteriaceae*, such as *Escherichia* and *Shigella* species [6]. It is also influenced by polyphenols, a group of bioactive compounds that are not absorbed in the blood throughout the small intestine and reach the colon, which reduce oxidative stress on gut microbes, give them a carbon source for metabolism, promote the formation of metabolites with antimicrobial action on pathogens like *Escherichia coli*, and the growth of specific microbes [6,7,8,9]. Dysbiosis is a disruption of the normal and dynamic crosstalk between the host and the microbes, as well as an incorrect balance between facultative anaerobic and aerobic bacteria; it has been clarified that dysbiosis is strongly associated with metabolic disorders like obesity and diabetes, cancer, and cardiovascular diseases. In particular, the pathogenesis of cancer is linked to a reduction in anti-inflammatory bacteria like *Lactobacillus*, *Roseburia*, *Akkermansia muciniphila*, and *Eubacterium*, and to an increase in the growth of proinflammatory species like *Ruminococcus gnavus* and *Bacteroidetes*. The mechanism at the base of dysbiosis is the break of gut-barrier integrity that promotes the translocation of bacteria fragments like lipopolysaccharide (LPS) to the blood, with consequences on systemic inflammation, oxidative state, cellular immunity, and metabolism (Figure 2) [19,20,21,22,23,24,25,26,27,28].

### 2.1. Modulation of NF-KB and IL-6/STAT-3 Pathway

The nuclear factor kappa-light-chain-enhancer of activated B cells (NF-KB) is a transcription factor involved in the regulation of the cell cycle, cell survival, and apoptosis; it normally ties to its antiapoptotic protein target B-cell lymphoma 2 (Bcl-2), leading to an augment of it and, as a consequence, to the inhibition of programmed death of cancer cells. Natural molecules, such as resveratrol (RES) and evodiamine (EVO), have crucial roles in the mechanisms of apoptosis. RES is a substance contained in peanuts, grapes, berries, red wine, and in plants after fungal infection, exposure to ultraviolet (UV) radiation, and mechanical injury, while EVO is an alkaloid found in *Evodia rutaescarpa*. They act by inhibiting NF-KB and consenting to Bcl-2 exerting its proapoptotic effect; in detail, EVO demonstrated its capacity to cause a decrease in NF-KB expression in a zymosan-induced inflammation model [20,29]. During inflammatory processes, immune cells produce interleukin 6 (IL-6) which ties to the soluble IL-6 receptor or the membrane IL-6 receptor and forms a complex that interacts with glycoprotein 130 and determines an upregulation of the transcription factor signal transducer and activator of transcription 3 (STAT-3); in the nucleus, STAT-3 stimulates the expression of molecules promoting cell proliferation and differentiation and antiapoptotic genes. A specific diet can cause a chronic increase in IL-6 levels, with the consequence that the intestinal mucosa becomes populated by constantly expanding and by apoptosis-resistant T cells but, at the same time, a change in diet permits the restoration of a healthy intestinal environment. Speaking of which, anthocyanins exert antioxidant effects, reducing the local amount of reactive oxygen species (ROS) and determining, as a consequence, a lower expression of IL-6 and a relief in inflammation-related stress on gut microbiota and epithelial cells; in an experiment with pigs, a high-calorie diet supplemented with purple potatoes containing anthocyanins led to a sixfold decrease of IL-6 levels compared to the diet without purple potatoes. In order to better understand the role of STAT-3 in gut-microbiota dysbiosis, researchers evaluated through immunohistochemical techniques the levels of phosphorylated STAT-3 (pSTAT-3) expression in eighteen colorectal cancer (CRC) samples and in the paracancerous tissues and demonstrated that more than 72% of CRC samples had an increased expression of pSTAT-3, especially localized in the nucleus. Similar results, with an upregulation of STAT-3, were obtained with Western Blot analysis; in other experiments, cells from CRC were incubated with an activator of STAT-3, colivelin, and specific measurements showed increased proliferation rates and levels of pSTAT-3, suggesting that the inhibitory action of EVO on CRC proliferation is due to the inhibitory activity of pSTAT-3 and that STAT-3 represents a connection between adverse immune-cell action and the development of a microenvironment promoting the activity of procancer cells, like myeloid-derived suppressor cells and tumor-associated macrophages [20,29].

### 2.2. Effects on Neoangiogenesis 

It is well known that neoangiogenesis is one of the most common mechanisms involved in the pathogenesis of cancer acting through the modulation of hypoxia-inducible factor (HIF) that, in turn, controls the synthesis of molecules such as vascular endothelial growth factor (VEGF), nitric oxide (NO), and angiopoetin-1. A recent study demonstrated that *Clostridium difficile* infection is able to determine an upregulation of VEGF and, consequently, promote vascular permeability and cancerogenesis [30,31,32,33,34,35,36]. Among natural compounds capable of influencing neoangiogenesis, evodiamine showed the ability to inhibit, in a dose-dependent manner, prostaglandin E2 and inducible nitric oxide synthase and cycloxygenase-2, with the result of a block on neovascularization [19]. Soybean contains a potent isoflavone called daidzein, which is metabolized by gut microbiota to form equol. This metabolite has higher biological activity than daidzein and exerts neuroprotective, anticancer, and antiangiogenetic effects; experiments showed that equol is able to downregulate VEGF expression and, consequently, reduce human endothelial cell growth from the umbilical vein. In fact, cells treated with equol showed lower levels of Ki67 and a lower expression of Cyclin D1/Cyclin E1 expression; in contrast, they were characterized by high levels of proapoptotic molecules like caspase-3, which promote the arrest of the cell cycle at the G0–G1 phase. This evidence indicates a possible role of daidzein and its metabolite equol in the induction of cancer cell death [36].

### 2.3. Antiproliferative and Proapoptotic Effects

*Vachellia tortilis* is a medical plant growing in semiarid and arid regions of Southern, Northern, and Eastern Africa, and in the Middle East. Its essential oils and extract were shown to have antibacterial activity in vitro due to the capability of cell-membrane disruption, including, for example, against multiresistant species of *Staphylococcus aureus*, *Pseudomonas aeruginosa*, and *Klebsiella*. Other in vitro experiments put into evidence a potent cytotoxic activity of this substance since, using a sulforhodamine B colorimetric assay, it was possible to show cell inhibition from nonsmall cell lung cancer, hepatocellular carcinoma, breast, and cervical cancer samples; elipticine was used in this study as a positive control and the results were expressed in terms of growth inhibition (GI) values. *Vachellia tortilis* extract determined a cytotoxic effect, especially on cells from hepatocellular carcinoma, which research attributed to the presence in it of epigallocatechin derivatives that, thanks to their high levels of hydroxyl ring substitutions, inhibit the proliferation of neoplastic cells. Furthermore, cancer cells were shown to be more reactive compared with normal cells to this natural substance [37,38,39,40,41,42,43,44]. Apoptosis seems to be stimulated also from *Propionibacterium* contained in dairy products, which produce the major part of short-chain fatty acids (SCFAs), in detail butyrate, acetate, and propionate. Experiments showed that SCFAs are capable of decreasing cell growth through a dual mechanism; they inhibit cell proliferation and increase apoptosis since a cell-cycle arrest can be observed after twenty-four hours in the first case and after forty-eight hours in the last one. Among processes used by cancer cells to spread all over the human organism, the Warburg effect is an adaption of neoplastic cell metabolism consisting of the use of glycolysis when there is a sufficient quantity of oxygen instead of the oxidative phosphorylation which should be used to satisfy their elevated energy needs. This effect is reflected by high levels of lactate in neoplastic cells, which has to be exported to the extracellular microenvironment to prevent acidification and cell death. The above evidence allows for the statement that SCFAs stimulate the production of energy throughout glycolysis, determining an overproduction of lactate. This condition could represent a potential therapeutic instrument because it would be possible to increase the amount of SCFAs in the diet through a major increase of dairy products, probiotics, nondigested proteins and fibers, all substances that modulate the composition of the gut microbiota, in terms of an increase in *Propionibacteria*, *Firmicutes*, *Bacterioidetes*, and *Fusobacteria*, which form SCFAs that, in turn, target colorectal cancer cells and preventing effects on normal cells [45,46].

### 2.4. Antiestrogenic Action

It is well known that elevated levels of estrogens cause interference with deoxyribonucleic acid (DNA) and lead to the formation of neoplastic cells. An incorrect production of estrogens could cause the conversion of 16α-hydroxylation to 16α-hydroxyestrone and the synthesis of 4-hydroxyestradiol, 2-hydroxyestradiol, 4-hydroxyestrone, and 2-hydroxyestrone; the oxidation of these molecules, with the consequent formation of estradiol-3,4-quinone and estrone-3,4-quinone, determines the synthesis of depurinating adducts through interaction with the host DNA, the formation of apurinic sites that cause DNA mutations, and, finally, the onset breast and prostate cancer. As explained above, daidzein is a soy isoflavone metabolized to equol from the gut microbiota. In detail, only 60% of Asian people and 30% of the Western population are able to synthetize equol, whereas the S-enantiomeric form of this molecule is produced through the degradation of daidzein from the gut microbiota, in particular microbes from the *Eggerthellaceae* family. Equol seems to be able to modulate the hormonal balance thanks to its bond to both estrogen receptors (ERs) ERα and ERß, blocking cell proliferation and inducing their apoptosis; the stimulation of programmed cell death involved caspase-7 and caspase-9, which are cleaved with the consequent release of cytochrome C in the cytosol, the effect on cytosolic targets, and, finally, apoptosis [36,47,48,49,50,51,52].

### 2.5. Modulation of Redox Signalling and Regulation of Foxp3+ T Cells

Experiments on colorectal cancer cells demonstrated that short-chain fatty acids, in particular butyrate, act at a nuclear level by inducing upregulation of genes for specific detoxifying enzymes like glutathione-S-transferases and exerting, consequently, primary cancer prevention. The effects of butyrate are dose dependent; in fact, at physiological concentrations, it induces cell-cycle arrest through mitochondria-mediated apoptosis and an increased mitochondrial superoxide/ROS formation, whereas medium and low levels induce cell differentiation and the block of the cell cycle at the G1 phase and, finally, high concentrations of butyrate, which causes massive apoptosis. Furthermore, this short-chain fatty acid modulates the proliferation of hepatic cells via miR-122 expression. In fact, it alters the mitochondrial membrane potential, causing downregulation of the NAD-dependent histone deacetylase Sirtuin-1 (HDAC SIRT-1) and leading to an increase in superoxide synthesis. As it is noted, miR-122 is downregulated and acts as a tumor suppressor in different types of cancer, like breast cancer, through an inhibition of cell migration, proliferation, and invasion; it would be very interesting to target miRNAs expression using natural substances in combination with traditional drugs in the treatment of cancer in order to potentiate the efficacy of conventional molecules and reduce the adverse effects [46,53,54]. Branched-chain amino acids (BCAAs), including leucine, isoleucine, and valine, are molecules with an aliphatic sidechain with a branch that cannot be produced by human organisms and must be introduced with food or be synthetized from gut microbes, specifically from *Prevotella copri* and *Bacteroides vulgatus*. Although BCAAs are known to be a supplement to increase muscle volume and reduce exercise fatigue, recent evidence has shown that elevated levels, deriving mainly from specific gut-microbiota compositions and from muscle proteins, are associated with systemic inflammation and metabolic disorders like diabetes and cancer. These molecules promote oxidative stress and the migration of blood mononuclear cells via mammalian target of rapamycin complex 1 (mTORC1) activation; in fact, BCAAs are essential in the mTORC1-dependent pathway responsible for cell proliferation. In mice, a diet with a low amount of BCAAs reduces the number and proliferation capacity of Foxp3+ regulatory T cells, determining globally a reduced signal for T-cell production in the thymus, incorrect homeostasis in the periphery, and unbalanced differentiation of cells into CD4+ Th2, Th1, Th17, and CD8+ T cells [20].

## 3. Different Types of Cancer

### 3.1. Hematological Malignancies

**Figure 3:**
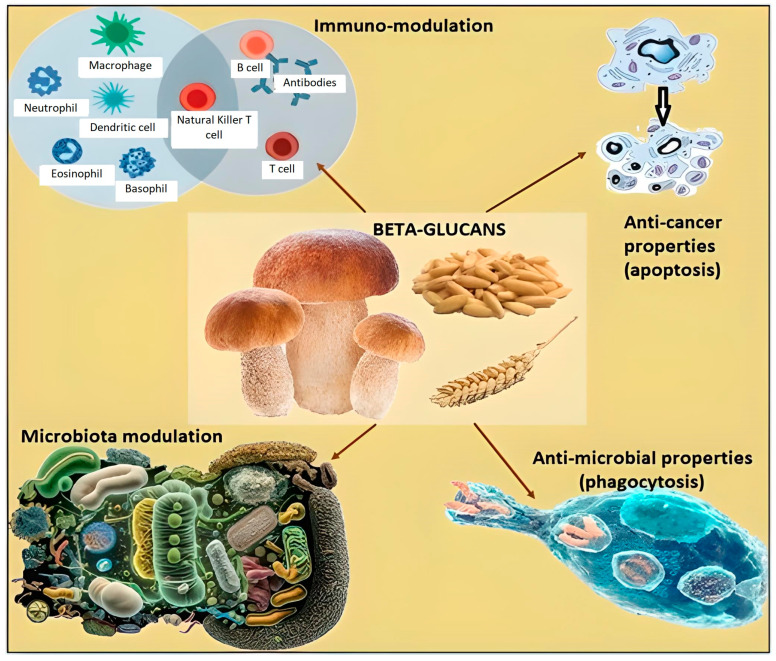
β-glucans contained in mushrooms, oat, and barley have several anticancer and antimicrobial effects and a pivotal role in microbiota and immune-cell modulation; in particular, it was demonstrated that Maitake mushroom extract led to a strong monocyte and neutrophil activity.

The biodiversity hypothesis states that reduced contact with plants causes negative effects on gut microbiota and, consequently, on the host’s immune response. Studies about hematological malignancies put in evidence that exposure to plant diversity and related microbial communities promotes immune-cell maturation and is linked to a reduced onset of childhood acute lymphoblastic leukemia (ALL). Acute lymphoblastic leukemia is the most common pediatric cancer, which develops in two steps. During pregnancy, a genetic mutation occurs and causes the formation of a preleukemic clone; after birth, a certain percentage of children, after external stressors like an infection, develop a secondary genetic mutation, leading to ALL. Even if the infection is the primum movens for the genetic change, good microbial exposure in the first years of the life of a child could contribute to a stronger immune system responding against infections and, consequently, prevent the trigger for genetic mutations. Even if children with ALL especially live in urban areas, have European ancestry, are less exposed to plant genera, and have fewer older siblings compared with children without ALL, more and more scientific experiences are necessary to state without any doubt that a link between plant diversity exposure and ALL onset exists [17]. Studies about hematological malignancies also involved beta-glucans (β-glucans), polysaccharides structurally formed by glucose molecules linked by beta-glycosidic linkages at diverse positions; they can be divided into two groups, called cereal and noncereal: cereal or grain includes barley, oat, rice, and wheat and contain 1,3 and 1,4 glycosidic linkages, while noncereal includes mushrooms, algae, bacteria, and yeast. β-glucans have shown various effects, including a reduction in cholesterol and glucose blood levels and a modulation of gut microbiota and immune response. So, they are being investigated as adjuvant substances both for hematological and solid malignancies, immune-mediated diseases like allergic rhinitis, and respiratory infections. The Food and Drug Administration (FDA), in 1997, approved oat bran, an important source of β-glucans, as the first food suitable to reduce cholesterol blood levels and recommended an ideal dose of 3 g/day, which corresponds to ≤60 g oatmeal or ≤40 g oat bran. In cancer, β-glucans are recognized as PAMPs and act in regulating complement-dependent cytotoxicity. In fact, in the bloodstream, they tie to endogenous anti-β glucan antibodies (ABA), leading to the activation of the complement system, which, through its component protein, iC3b ties to ABA, with a resulting β-glucans–ABA–iC3b complex. The last binds to immune effector cells and stimulates phagocytosis. Specifically, it leads to a direct killing of antibody-targeted neoplastic cells. In a preclinical model, mice received monoclonal antibodies and β-glucans, with the result of a strong regression of both hepatic and mammary tumors. Preclinical studies in mice, after intraperitoneal injection of *Escherichia coli*, demonstrated which animals treated with β-glucans reached zero in bacterial counts from peripheral blood, while those that did not receive β-glucans had a mortality rate of 100% after twenty-four hours [55]. Furthermore, the administration of β-glucan to rats with sepsis led to the reaching of benefits in terms of the reduction of proinflammatory cytokines like IL-6 and an increase in anti-inflammatory ones, with positive consequences on the infectious process. In vivo research involving patients with myelodysplastic syndromes demonstrated that Maitake mushroom extract augmented monocyte and neutrophil activity; moreover, a phase I clinical trial enrolling twenty patients with chronic lymphocytic leukemia was developed and showed that combined treatment with monoclonal antibodies β-glucans was well tolerated. So, it has been administered also in phase II of the study. The concept of this evidence is that β-glucans could augment the cytotoxic activity of the innate immune system if administered together with monoclonal antibodies and represent a possible instrument to fight cancer in combination with traditional strategies (Figure 3 and Table 1) [56].

Methotrexate (MTX) is an antimetabolite of folic acid used as an inhibitor of dihydrofolate reductase (DHFR) in the treatment of autoimmune and neoplastic diseases; it is unfortunately associated with adverse effects on multiple organs and this fact represents an important cause of drug withdrawal. In a microbiome and metabolomic profiling study involving rats treated with MTX, scientists put in evidence changes in fecal metabolites and gut bacterial changes in a dose-dependent manner and at different periods of time after MTX administration. In detail, an abundance of Firmicutes over Bacteroidetes was observed when using low doses, while a reverse trend when using high doses. Furthermore, experiments suggested the role of dysregulated gut microbiota in the development of intercellular connections. In fact, MTX induces an altered expression of Claudin-1, E-cadherin, and other cell-adhesion proteins. These effects could be overpassed by using adjuvant therapies in order to restore a correct balance in gut microbiota. For example, folic acid could be supplemented with the aim of preventing folate deficiency for healthy tissues and reducing the rate of adverse effects, especially when using high doses of MTX in hematological malignancies treatment. In vivo studies testing MTX toxicity in rats showed that yogurt fermented with Lactobacillus bulgaricus, together with Streptococcus thermophilus, and fermented with Lactobacillus johnsonii were able to prevent MTX damage to the small intestine through an improvement of intestinal barrier function [57,58,59,60,61,62,63,64,65].

**Table 1:** Clinical trials about the use of natural compounds influencing gut microbiota in hematological malignancies (clinicaltrials.gov accessed on 8 June 2023) [65].

Study Title	Conditions	Study Type	Phase	NCT Number
Rituximab Plus Beta-Glucan in Chronic Lymphocytic Leukemia (CLL)/Small Lymphocytic Lymphoma (SLL)	Chronic Lymphocytic Leukemia; Small Lymphocytic Lymphoma	Interventional	Phase 2	NCT00290407
Imprime PGG, Alemtuzumab, and Rituximab in Treating Patients With High Risk Chronic Lymphocytic Leukemia	B-cell Chronic Lymphocytic Leukemia; Refractory Chronic Lymphocytic Leukemia; Stage 0 Chronic Lymphocytic Leukemia	Interventional	Phase 2	NCT01269385
A Phase 2 Clinical Trial of Rituximab and B-Glucan PGG in Relapsed Indolent Non-Hodgkin Lymphoma	Relapsed/Refractory Indolent B Cell Non-Hodgkin Lymphomas	Interventional	Phase 2	NCT02086175

### 3.2. Colon Cancer

High amounts of alcohol, fat, and red meat in the diet, together with low fiber consumption, are associated with an augmented risk of colorectal cancer. In fact, these wrong food habits cause microbial dysbiosis consisting of a reduced rate of symbionts bacteria, which are substituted by procarcinogenic and inflammation-related microbes; *Escherichia coli*, *Streptococcus gallolyticus*, *Enterococcus faecalis*, *Bifidobacterium*, *Lactobacillus*, and *Campylobacter* promote the transformation of adenomas, the precursors of CRC, in adenocarcinomas via invasion through a breach in the colon epithelial barrier [28]. In a mouse model evaluating the pathogenesis of colon cancer, after a treatment with evodiamine, mice showed changes in gut microbiota resulting in an increased expression of Bifidobacterium, Campylobacter, and Lactobacillus and a reduced expression of Enterococcus faecalis and *Escherichia coli*. These data put in evidence the pivotal role of EVO in modulating gut microbiota in a disturbance sense for colorectal cancer in mice [19]. Nature offers other substances with proven effects in preventing CRC, such as *Scutellaria baicalensis*, an herb used in the Oriental medical system for its antioxidant and anti-inflammatory properties; it contains some flavonoids like baicalin (BI), wogonoside, baicalein (BE), and wogonin. In vitro studies showed that baicalin and wogonoside from *Scutellaria baicalensis* extract incubated with gut microbiota were converted into baicalein and wogonin that, in turn, exerted anticancer effects in a colon cancer mouse model. In a phase I clinical trial enrolling healthy rats, a dose of 100–2800 mg of baicalein was administered and it was demonstrated to be well tolerated and safe; moreover, the survival rate was thirty-six weeks compared with that of the model group of twenty weeks. So, it can be stated that the average life span increased by about 75% [66,67,68,69,70,71,72,73,74,75]. The human diet also allows for the intake of polyphenols that have a small absorption in the small intestine. In fact, 90% of unabsorbed molecules accumulate in the large intestine together with conjugated bile and they are exposed to the action of gut microbiota enzymes which, in turn, convert polyphenols into bioavailable compounds of lower molecular weight as phenolic acids. For example, resveratrol is converted into piceid, and the proanthocyanidins 3-Hydroxyphenylpyruvic acid into an anthocyanidin with crucial roles in reducing inflammation processes through the NF-KB pathway and oxidative stress induced by low-density lipoproteins (LDL). Gallic acid (GA) showed an important capability of regulation of mitochondrial functions through the activation of 5′ AMP-activated protein kinase (AMPK) and peroxisome proliferator-activated receptor gamma coactivator 1-alpha (PGC1α) [20,54]. Green tea derives from the leaves of Camellia sinensis infused to obtain this beverage, which is one of the most consumed all over the world. It is well known for its anti-inflammatory and antioxidant properties against diabetes, obesity, and cancer, with some of these benefits connected to the interplay between green-tea compounds and gut microbiota. In fact, these natural substances stimulate specific gut microbes with positive effects on the human organism. Green tea is constituted of 24–36% of phenolic compounds (especially catechins), 15% of proteins, and 7% of lignin, followed by small percentages of caffeine, amino acids, chlorophyll, and organic acids. The catechins act in a dual modality, stimulating healthy gut microbes and inhibiting the proliferation of *Campylobacter jejuni*, *Bacillus cereus*, *Escherichia coli*, *Clostridium perfringens*, *Legionella pneumophila*, and *Helicobacter pylori* through a disruption of the cell membrane. Furthermore, green-tea polyphenols are able to create an overproliferation of *Faecalibacterium*, *Bifidobacterium*, *Eubacterium*, and *Roseburia* that lead to an increase in SCFAs with proven effects against colorectal cancer because they suppress lipopolysaccharide synthesis and, consequently, potentiate immune system function and exert an anti-inflammatory action [76,77,78,79,80,81,82]. Citrus fruits, like *Citrus reticulate*, or mandarin oranges; *Citrus sinesis*, or sweet oranges; *Citrus depressa*, or flat lemons; *Citrus aurantium*, or bitter oranges; and, especially, *Citrus tangerine*, or tangerines, contain nobiletin, a flavone with a chemical structure of three aromatic rings, each one linked to specific molecular groups. After long-term storage, nobiletin goes towards the autohydrolysis process, determining its degradation into 5-demethylnobiletin. The last compound can be also obtained through the conversion of orally consumed nobiletin by gastric acid. Both the initial molecule and its derivate have shown, in vitro, interesting cytotoxic properties against colon cancer cells consisting of cell-cycle block and the induction of programmed cell death. Another mechanism through which nobiletin acts is its capacity to antagonize carcinogens like 2-amino-1-methyl-6-phenylimidazo[4,5-b]pyridine and azoxymethane. In a mouse model, the administration of a compound containing 0.01% of nobiletin for five weeks led to a regulation of azoxymethane-related altered cell proliferation with the result of a 50% reduction in treated mice compared with the control group; in particular, nobiletin was showed to be able to reduce the proliferation rate by 69% and downregulate IL-6, TNF-α, and IL-1β in treated mice [19].

### 3.3. Breast Cancer

Green-tea polyphenols and broccoli sprouts have been studied to understand their possible role, alone and in combination, in breast cancer pathogenesis. Experiments in a mouse model about an estrogen-receptor (ER) negative mammary tumor showed that polyphenols alone did not induce a significative change in the gut-microbiota composition of healthy mice, even if they reduced significantly the rate of Firmicutes communities; in contrast, after cancer onset, these natural compounds effectively modified gut microbes, leading to a reduction of Proteobacteria and an increase in Bacterioides, compared to the control group. Furthermore, these changes are more evident after a combined administration of both broccoli sprouts and green-tea polyphenols [76,77,78,79,80,81]. Other plants and vegetables have shown antioxidant and anticancer properties. Lycopene, a carotenoid found in red fruits and vegetables like tomatoes, acts through the regulation of growth factor signals, induction of cell-cycle block, and stimulation of apoptosis; in vitro studies involving ER-positive breast cancer proved that lycopene upregulated the expression of the proapoptotic protein Bcl-2 and of the suppressor p53 suppressor. Quercetin, a flavonoid found in vegetables and fruits, shows pro-oxidant and antioxidant properties, depending on the redox state of cells and on its concentration; it acts to suppress the calcineurin pathway activation with a final inhibition of angiogenesis. Moreover, a synergistic action of quercetin and vitamin C has been evidenced in reducing the expression of NRF2-specific messenger ribonucleic acid (mRNA), together with its nuclear translocation and protein synthesis [46].

### 3.4. Prostate Cancer

Prostate cancer is the most frequent tumor in males in Western countries. In vivo studies tested the administration of one or more portions of broccoli weekly and demonstrated that this strategy could reduce the incidence of the disease and the development of invasive and aggressive forms deriving from localized ones. A role in this reduction of incidence is due to the glutathione S-transferase mu 1 (GSTM1) genotype. Specifically, the expression of one allele (about 50% of the population) represents a protective factor respecting the homozygous deletion of the gene. Broccoli contains 3-methylsulphinylpropyl and 4-methylsulphinylbutyl glucosinolates in the florets. These molecules are converted into iberin (IB) and sulforaphane (SF) by plant thioglucosidases after tissue damage or by microbial thioglucosidases of the colon if food has been cooked or frozen. When in the human intestine, iberin and sulforaphane are absorbed by enterocytes and conjugated with glutathione, finally passing into the blood where they are metabolized through the mercapturic acid system and eliminated as -acetylcysteine conjugates with urine. Sulforaphane has a high bioavailability in humans with a plasmatic peak of four hours. After oral administration, it spreads in all human tissues, even if in different proportions (low levels in the central nervous system). It is absorbed and conjugated inside cells with glutathione; then, it is excreted after four hours from the liver, brain, and kidneys and, after twenty-four hours, from other organs. It acts as an inhibitor of histone deacetylase, an enzyme involved in gene expression. Specifically, its metabolite sulforaphane-acetylcysteine seems to be able to activate mitogen-activated protein kinases (MAPKs) and inhibit the response of neoplastic tissues to androgens, with a global anticancer effect. Furthermore, sulforaphane has anti-inflammatory properties because of its capability to block NF-KB translocation to the nucleus and, consequently, impede the cascade of processes leading to proinflammatory cytokines synthesis [82]. Gene-expression profiles from human prostate biopsies differ from before, during, and after a year of assumption of a diet rich in broccoli and peas. Experiments demonstrated that, after this diet-based treatment, 45% of sulforaphane is present as free SF in plasma and its peak concentration is less than 2 mM, with low levels after a few hours. Moreover, the excretion of sulforaphane through the mercapturic acid pathway is higher in GSTM1 null men, with the remaining amount of SF metabolized via an unknown system. This fact could give an explanation for the anticancer effect of broccoli. In vitro and in vivo evidence shows which SF is an important inducer of transcription of specific genes for phase-two enzymes and for molecules involved in the stimulation of cell-cycle block and programmed cell death; it is important to specify that these effects occur after an exposition of cells to very high levels of sulforaphane, like 100 mM for 24 h, with crucial differences in the concentration of SF in the blood after a single consumption of broccoli. In fact, at physiological concentrations, all sulforaphane entering cells is rapidly conjugated with glutathione thanks to the high levels of this last molecule in cells [83].

### 3.5. Hepatocellular Carcinoma

Hepatocellular carcinoma (HCC) is an aggressive tumor with a poor prognosis and a high rate of mortality. Among the risk factors, infections and a wrong lifestyle, including smoking, alcohol consumption, and exposition to polycyclic aromatic hydrocarbons and toxins like aflatoxin 7, represent the most important ones. A randomized, placebo-controlled study that used oltipraz and chlorophyllin proved an important reduction of aflatoxin-7 in urine and augmentation of its metabolite aflatoxin-mercapturic acid. This condition was associated by scientists with a consistent protection against HCC in animal models; it was hypothesized that these molecules act thanks to their capability to stimulate the Keap1-NRF2 pathway and, consequently, induce phase 2 gene expression. Studies about hepatocellular carcinoma involving natural compounds with the capacity to modify, in a positive sense, gut microbiota evaluated the effects of broccoli sprouts both in preclinical and clinical trials. They are an exceptional source of sulforaphane and are different from the mature plant and are available in markets because broccoli sprouts contain fifty times more glucoraphanin, the precursor of sulforaphane, per gram of fresh aliment and do not have h-hydroxyalkenyl glucosinolates and indole, linked to a potential toxic effect. The substance to administer derives from broccoli sprouts maintained in boiling water with the result of a good extraction of glucoraphanin and the production of a beverage with defined contents of this molecule and suitable to be frozen and, then, easily distributed to people enrolled in the intervention. This method, however, causes the inactivation of myrosinase, the plant enzyme responsible for the hydrolysis of glucoraphanin to sulforaphane, impeding the activation of glucosinolates to the active isothiocyanates. At the same time, it is of note that the bioavailability of sulforaphane from a boiled extract completely depends on gut microbiota for hydrolysis. In fact, an important interindividual variability has been observed in glucoraphanin pharmacokinetics and it could reflect the different composition of enteric microbiota influencing polymorphisms in genes involved in dithiocarbamate production and the amount of glucoraphanin hydrolysis. Nonetheless, this evidence opens the road to a possible rational and practical use of food-derived molecules as chemopreventive agents [84,85,86,87,88,89,90,91,92,93,94,95,96,97,98,99].

## 4. Conclusions and New Perspectives

**Figure 4:**
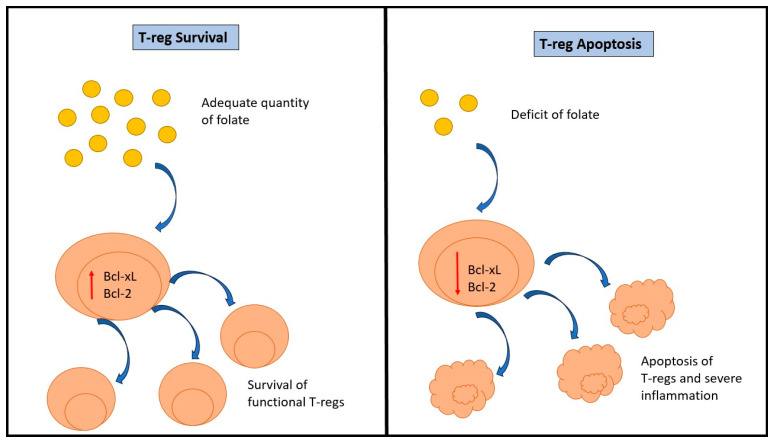
An inappropriate amount of folate causes apoptosis of T-regs and, consequently, reduced immunosuppression and severe inflammation.

The gut microbiota is a dynamic community made up of trillions of bacteria involved in crucial functions in intestinal homeostasis and digestion; it changes both in a positive and negative sense for human health in response to diseases, diet, use of antibiotics and probiotics, hygiene status, and other environmental factors. A bidirectional crosstalk between the immune system and gut microbiota is fundamental for the attainment of adaptive and innate immune responses. In fact, intestinal microbes influence the development of lymphoid tissues, promote local mucosal immunity regulation thanks to direct contact with immune cells, and support lymphocyte subpopulations in secondary lymphoid organs. Immune cells constituting the gut microbiota are different in location and frequency in the intestine. The intestinal epithelium of the small intestine is the richest in CD8+ T cells, which can be divided into T regulatory cells (Tregs) and T helper (Th); 17 are the most abundant, with the first mainly present in the colon while the second in the proximal small intestine. Moreover, gut lamina propria contains B cells, T cells, innate lymphoid cells, dendritic cells, and natural killer cells. If a disruption of the normal balance between the host and the microbes occurs, the so-called dysbiosis, there is an increase in the risk of metabolic disorders like obesity and diabetes, cancer, and cardiovascular diseases. In particular, the pathogenesis of this pathological condition is linked to a reduction of anti-inflammatory bacteria like *Lactobacillus*, *Roseburia*, *Akkermansia muciniphila*, and *Eubacterium*, and an increase in the growth of proinflammatory species like *Ruminococcus gnavus* and *Bacteroidetes*. The mechanism at the base of dysbiosis is the break of gut-barrier integrity that promotes the translocation of bacteria fragments like lipopolysaccharide (LPS) to the blood, with consequences of systemic inflammation, oxidative state, cellular immunity, and metabolism. A diet rich in vegetables has several benefits in terms of health promotion and is linked to a reduction in systemic and local intestinal inflammation. For example, resveratrol is a substance contained in peanuts, grapes, berries, and red wine, in plants after fungal infection, exposure to ultra-violet (UV) radiation, and mechanical injury, and acts by inhibiting NF-KB and consenting Bcl-2 to exert its proapoptotic effect [20,28,55,100,101,102,103,104,105,106,107,108,109]. During inflammatory processes, immune cells produce interleukin 6 (IL-6) which ties to the soluble IL-6 receptor or the membrane IL-6 receptor and forms a complex that interacts with glycoprotein 130 and determines an upregulation of the transcription factor signal transducer and activator of transcription 3 (STAT-3). In the nucleus, STAT-3 stimulates the expression of molecules promoting cell proliferation and differentiation and antiapoptotic genes. A specific diet can cause a chronic augment of IL-6 levels with the consequence that the intestinal mucosa becomes populated by constantly expanding and apoptosis-resistant T cells. But, at the same time, a change in diet permits the restoration of a healthy intestinal environment. Speaking of which, anthocyanins exert antioxidant effects, reducing the local amount of reactive oxygen species (ROS) and determining, as a consequence, a lower expression of IL-6 and a relief in inflammation-related stress of gut microbiota and epithelial cells. In an experiment with pigs, a high-calorie diet supplemented with purple potatoes containing anthocyanins led to a sixfold decrease in IL-6 levels compared to the diet without purple potatoes. Different diseases, in terms of relationships between gut-microbiota modulation and foods, and research about the polysaccharides β-glucans in hematological malignancies allowed for the proving of which of these molecules are recognized as PAMPs by the human organism and act regulating complement-dependent cytotoxicity [109]. In fact, in the bloodstream, they tie to endogenous anti-β glucan antibodies (ABA), leading to the activation of the complement system, which, through its component protein iC3b, ties to ABA, with the result of a β-glucans–ABA–iC3b complex. The last binds to immune effector cells and stimulates phagocytosis. In detail, it leads to a direct killing of antibody-targeted neoplastic cells. This straight interconnection between gut microbiota and the immune system allows immune cells to develop in a correct way and promotes the attainment of the immune balance. A phase II clinical trial about patients with chronic lymphocytic leukemia put in evidence the capability of β-glucans to potentiate the immune system activity if administered in combination with monoclonal antibodies; another in vivo experiment showed that Maitake mushroom extract determined an augment in neutrophil and monocyte functions in patients with myelodysplastic syndromes. Furthermore, preclinical studies in mice treated with an intraperitoneal injection of *Escherichia coli* demonstrated that animals treated with β-glucans reached zero in bacterial counts from peripheral blood, while those that were not treated with β-glucans had a mortality rate of 100% after twenty-four hours [55]. In this context, micronutrients such as vitamins are fundamental; in fact, vitamin B6 or pyridoxine regulates the homeostasis of T cells in differentiating into Th1/Th2 cells, with obvious and positive consequences in terms of cellular and humoral immunity development. A deficiency of vitamin B6 causes a reduction in CD8+ cell proliferation and activation through the suppression of the Th1 immune response and the stimulation of the Th2 response. A downregulation of Th1 function leads to a reduced interferon–gamma synthesis and the development of a delayed-type of hypersensitivity together with a lower capacity of T lymphocytes to maintain immunosurveillance against pathogens and cancer cells. Moreover, overstimulation of the Th2 response causes excessive production of type 2 cytokines such as interleukin-4 (IL-4), interleukin-13 (IL-13), and interleukin-5 (IL-5), with results in terms of proinflammatory responses. In the homeostasis of the immune system, folate is involved also since it stimulates Treg function and survival. Its role is marked by the high expression of folate receptor (FR4) present on the surface of Tregs. Different studies put in evidence the capability of folate in blocking FR4, leading to an important reduction of the antiapoptotic proteins B-cell lymphoma 2 (Bcl-2) and B-cell lymphoma-extra large (Bcl-xL) and, consequently, to an increase in the apoptosis of Tregs (Figure 4).

A deficit of folate not only has consequences on gut dysbiosis and Treg immune profiles but influences also the response to treatment with the so-called immune-checkpoint inhibitors (ICIs), drugs used in cancer, and targeting specific molecules expressed by neoplastic cells. Moreover, there are negative effects in terms of adverse events related to ICIs like a cascade of inflammatory events throughout the body, especially in organs targeted in autoimmunity, such as the intestine, with the result of severe inflammation due to the dysregulation of Tregs and the augmented risk of autoimmune disease onset [109,110,111,112,113,114,115,116,117,118,119,120]. Different clinical and preclinical trials studied ICI response in relation to the composition of gut microbiota and put in evidence that specific bacteria like Akkermansia and Bifidocterium are elevated in patients responding to immunotherapies versus nonresponders. It represented the point of start for successive experiments in which oral supplementation of Bifidobacterium in mice with cancer induced an enhancement in CD8+ T-cell development and improved immune-checkpoint inhibitor efficacy; furthermore, human studies about fecal microbiota transplantation (FMT) from immune-checkpoint inhibitor responders determined a better anticancer response compared with that of FMTs from nonresponder subjects. In particular, a very interesting clinical trial showed that fecal microbiota transplantation from responders to treatment with anti-PD1 led to a reorganization of the tumor microenvironment with positive results also in terms of overcoming resistance to PD-1 block in a group of patients with melanoma. Response rates seem to be linked also to the amount of vitamins. In fact, research has described vitamin B6 as a natural immunoregulator because it induces T-cell subtyping, stimulates PD-1/PD-L1 block, reduces the incidence of adverse events related to immune-checkpoint inhibitors, and is a potential therapeutic instrument to be used in combination with immunotherapy [121,122,123,124,125,126]. The data presented in this review just represent a point of start for future research with the aim, for example, to improve the extraction methods of molecules from plants and food, overcoming the possible contamination of the compounds with other natural molecules useless or toxic for the treatment. Although all the evidence presented in this work is very interesting, the possibility of using natural substances to influence the gut microbiota, in an anticancer sense, is discussed and more studies should clarify, without any doubt, the mechanisms of action of natural molecules, the response of gut microbes to them, and the consequences on onset, progression, and response to therapy of tumors, with the hope to have in the future new effective instruments to use in daily clinical practice against cancer.
